# Supplementary material for: THAP9 Transposase Cleaves DNA via Conserved Acidic Residues in an RNaseH-Like Domain
Source: Cells. 2021 May 29;10(6):1351. doi: 10.3390/cells10061351 (PMC8230255; doi:10.3390/cells10061351)
Supplement: Supplementary file 1 [file cells-10-01351-s001.zip › cells-1197053-supplementary.pdf]

Supplementary Information

This PDF file includes: Figures S1 to S7, Table S1

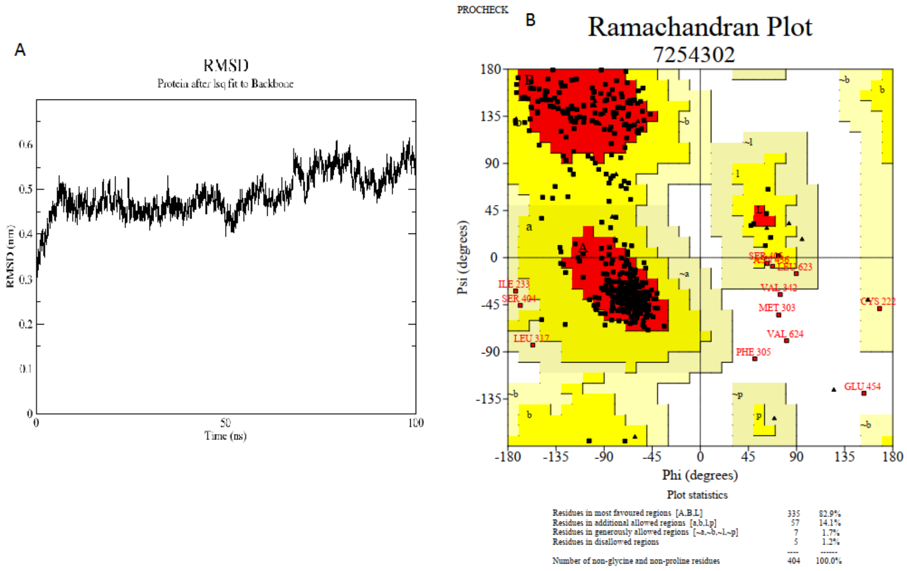

Fig S1: RMSD plot and Ramachandran plot for hTHAP9 model after MD simulations.

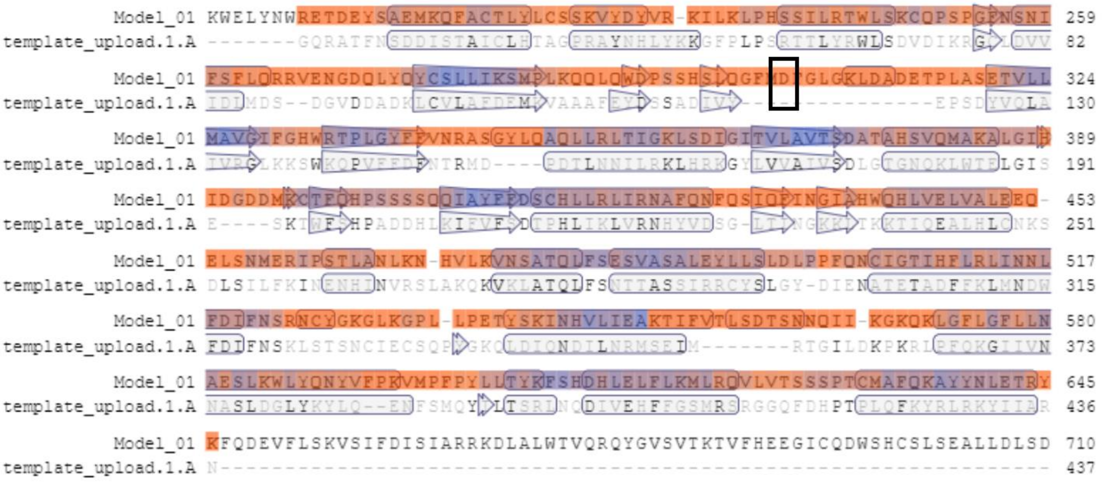

Fig S2: SWISS MODEL secondary structure alignment of hTHAP9 (Model_01) homology structure built with DmTNP template (template_upload.1.A). The residues enclosed in horizontal bars represent helical secondary structure and residues enclosed in arrows represent beta sheets. The colour of the sequence represents QMEAN, orange to blue indicates low to high quality estimate (23). D304 of hTHAP9, highlighted in black box, does not align to any structure and ends up in a loop in the final structure. Residue numbers correspond to the amino acid position in the primary sequence of the protein

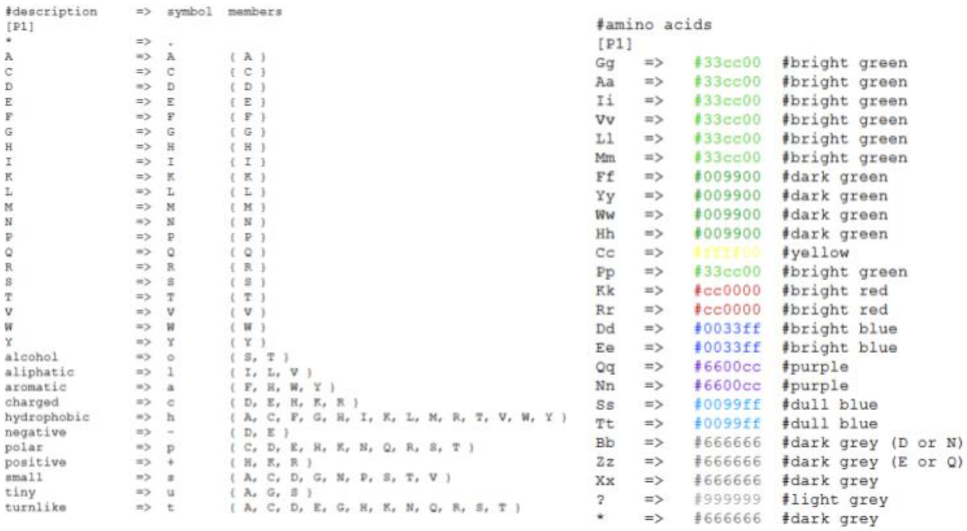

Fig S3: P1 colour palette for multiple sequence alignment viewed in MView.

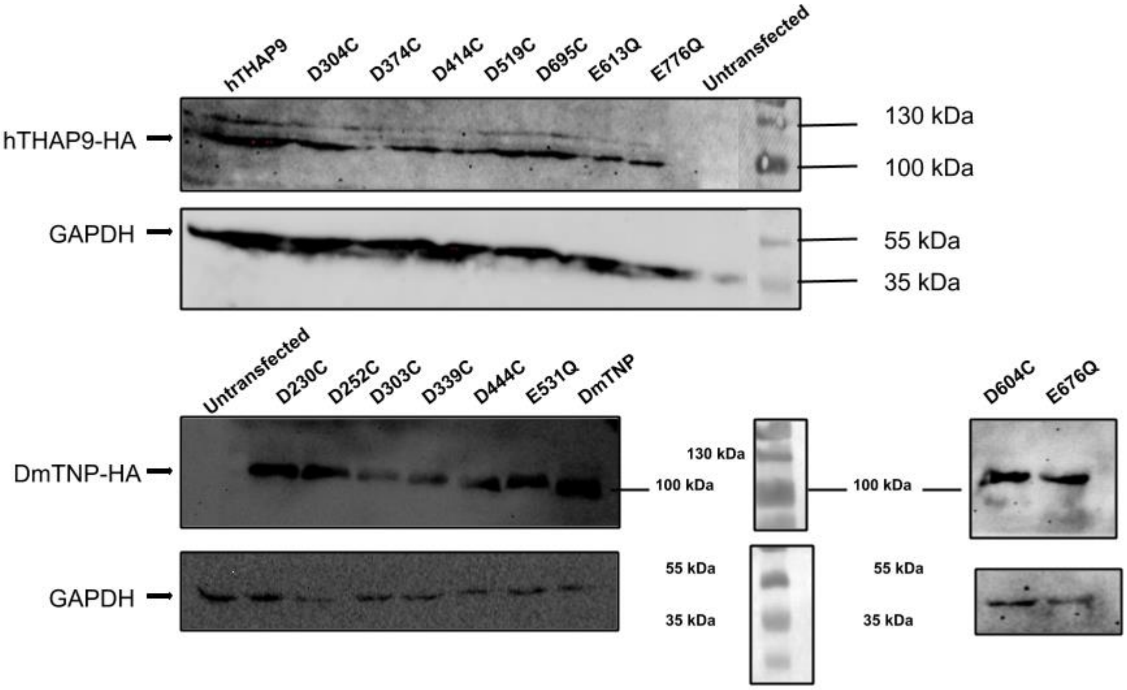

Fig S4: Expression levels of mutant and wild-type hTHAP9 and DmTNP (~100 kDa, C-terminal HA tagged) in the DNA excision assay as shown by western blot analysis (anti-HA). GAPDH (mol weight ~36 kDa) was used as a loading control. Negative control (HEK293 cells alone)

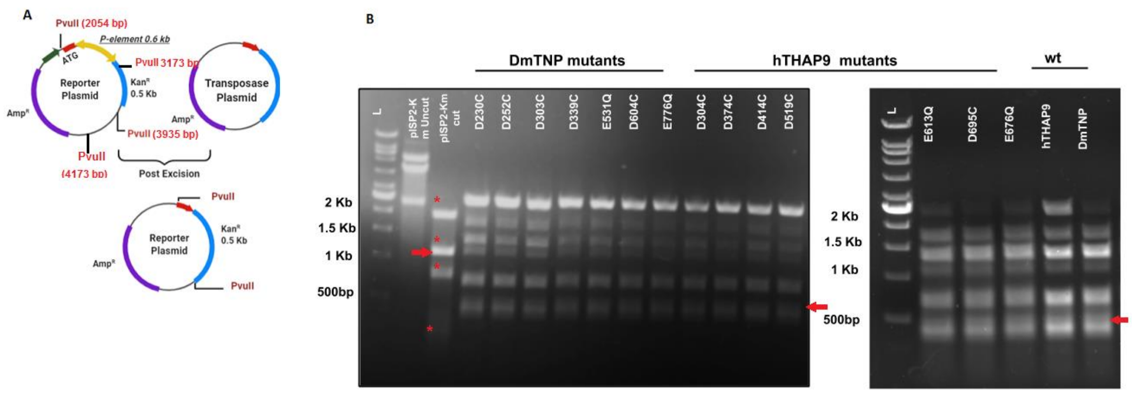

Fig S5 (A) Schematic representation of PvuII digestion assay of recovered reporter plasmid (obtained after transformation of bacteria with plasmid DNA isolated from HEK 293 cells transfected with DmTNP, hTHAP9 or the acidic residue mutants) (B) Agarose gel image of reporter plasmid after restriction digestion with PvuII enzyme. Diagnostic bands (1.1kb, 500bp are marked with red arrows)

A. **P element insertion site in pISP2/Km**

2232 **8bp TSD**

ATGACCATGATTACGAATTCCCGGGGATCTGCCGCTGGACTACGTGG **GTCTGGCC**

17nt P element 5' end **0.6 kb** **17nt P element 3' end** **8bp TSD**

CATGATGAAATAACATA- Pelement- TATGTTATTTCATCATG **GTCTGCC** ATTCTCAT

B. **Donor site repair**

ATGACCATGATTACG---CACTGGCCGTCGTT

2232 4088

Δ 40 upstream Downstream rearrangement

Fig S6. **DNA sequence analysis of P element excision products** (A) Schematic of P element insertion site within pISP2/Km plasmid. The 8bp target site duplications (TSDs) flanking the P element site insertion (red) are underlined and bold. The underlined ATG, upstream of P element insertion is the start codon of LacZ gene in pISP2-Km (20). (B) Typical pattern of donor site repair junction obtained by sequencing pISP2-Km plasmids recovered from individual kanamycin-resistant bacterial colonies (both wildtype and mutant DmTNP and hTHAP9). The red dashed lines represent the excised P element. Blue numbers represent the nucleotide position within pISP2/Km reporter plasmid. It is observed that the P element excision and subsequent repair often involve DNA

rearrangements and extensive deletions, as observed before (16).

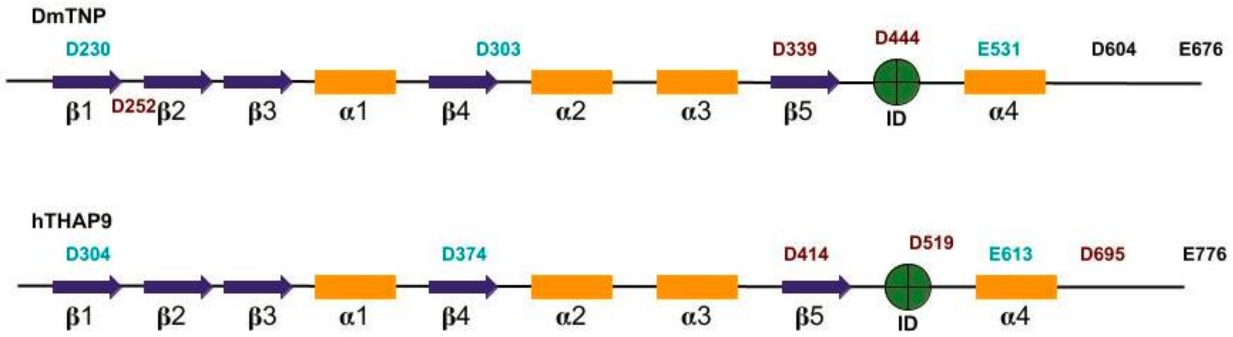

Fig S7: Summary of catalytic mutants in Drosophila and human THAP9. DDE motifs for each homolog are represented in bold (cyan) above its putative location. Other conserved residues mutated for the study are marked in black and brown (lower excision activity). Blue arrows indicate beta sheets and yellow bars represent alpha helices. Insertion domains (ID) for each homolog [DmTNP (residues 339-528) (12), hTHAP9 (residues 415-604), Pdre2 (residues 475-670) are shown in green circles.

Table S1: Relative DNA excision and DNA integration activities observed in plasmid-based assays.

	Mutants	% Relative excision activity	% Relative Integration activity
DmTNP	D230C	12.7	19
	D252C	7.83	31
	D303C	14.02	18.33
	D339C	23.65	76.33
	D444C	0	7.33
	D604C	12.64	63.33
	E531Q	10.99	3
	E676Q	49.6	76.66
	WT DmTNP	100	100

	pISP2-Km	0	-
	Cg4-Neo	-	8
	pCDNA 3.1	-	0
	pBluescript	-	0
**hTHAP9**	D304C	18.19	27
	D374C	26.07	5.5
	D414C	58.03	43.5
	D519C	10.26	14
	E613Q	10.26	7.5
	D695C	141.47	55
	E776Q	94.75	144.5
	WT hTHAP9	100	100
	pISP2-Km	0	-
	pBluescript	-	0
	Cg4-Neo	-	8
	pCDNA 3.1	-	0
